# A rare cause of neonatal respiratory distress: Jeune syndrome

**DOI:** 10.1016/j.radcr.2026.05.059

**Published:** 2026-06-11

**Authors:** Salma El Aouadi, Soukaina Bahha, Ouiam Taibi, Rania Bouanane, Nazik Allali, Latifa Chat, Siham El Haddad

**Affiliations:** Mother–Child Imaging Department, Ibn Sina University Hospital Center, Mohammed V University, Rabat, Morocco

**Keywords:** Jeune syndrome, Asphyxiating thoracic dystrophy, Neonatal respiratory distress, Skeletal dysplasia

## Abstract

Asphyxiating thoracic dystrophy, also known as Jeune syndrome, is a rare skeletal dysplasia characterized by a constricted thoracic cage and variable skeletal abnormalities that may result in severe respiratory insufficiency in the neonatal period. We report the case of a term male neonate admitted on the first day of life for acute respiratory distress. Imaging revealed a markedly narrow thorax with short ribs and elevated clavicles, associated with characteristic pelvic abnormalities including trident-shaped acetabula and short iliac bones, consistent with Jeune syndrome. Despite intensive supportive care and mechanical ventilation, the clinical course was complicated by progressive respiratory failure, resulting in death after 7 days. This case illustrates the characteristic imaging findings of Jeune syndrome and emphasizes the role of imaging in establishing the diagnosis.

## Introduction

Jeune syndrome, also known as asphyxiating thoracic dystrophy, is a rare autosomal recessive skeletal ciliopathy characterized by a markedly constricted thoracic cage associated with variable skeletal abnormalities and possible multiorgan involvement [1]. The reduced thoracic volume limits pulmonary expansion and frequently leads to severe respiratory compromise in the neonatal period [2]. Given its rarity and clinical variability, recognition of the characteristic imaging findings is essential for establishing the diagnosis [1]. We report a case of Jeune syndrome in a neonate presenting with severe respiratory distress, highlighting the key radiological features that facilitate early diagnosis of this rare and potentially life-threatening disorder.

## Case report

A male neonate, aged 1 day, born at term following an uneventful pregnancy, was admitted to the neonatal intensive care unit for acute respiratory distress. The patient was born to first-degree consanguineous parents. The mother was aged 34 years, gravida 3 para 3, with no significant obstetrical history. Prenatal follow-up was regular, and no fetal abnormalities were detected on antenatal ultrasonography. Birth weight was 3200 g, 49 cm, and Apgar scores were 5 and 7 at 1 and 5 minutes, respectively.

On admission, the newborn was tonic and reactive. Respiratory examination revealed marked tachypnea with an oxygen saturation of 79% on room air, associated with severe signs of respiratory distress, including pronounced intercostal and subcostal retractions, nasal flaring, and a narrow, constricted thoracic cage (Fig. 1). Cardiovascular and abdominal examinations were otherwise unremarkable.

**Fig. 1:**
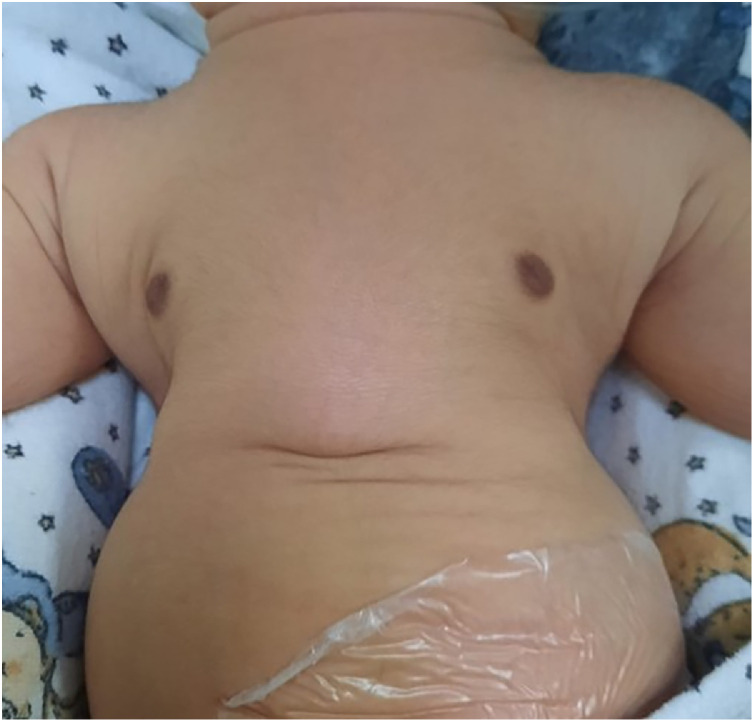
Clinical image of the neonatal patient showing a thoracic deformity with a markedly constricted thoracic cage.

Due to worsening respiratory failure, the patient was transferred to the neonatal intensive care unit and required endotracheal intubation and mechanical ventilation. Postintubation oxygen saturation improved to 93% under ventilatory support. Despite initial stabilization, lung compliance remained poor, consistent with a restrictive ventilatory pattern related to chest wall abnormality.

A chest radiograph obtained at admission demonstrated a short, narrow, elongated thoracic cage with reduced transverse diameter, high-riding clavicles with a characteristic “handlebar” appearance, and irregular costochondral junctions, raising suspicion for an underlying skeletal dysplasia (Fig. 2). Thoracic computed tomography performed to identify the cause of respiratory distress confirmed the severe thoracic narrowing with short, horizontally oriented ribs and elevated clavicles. Additionally, bilateral pulmonary consolidations consistent with infectious pneumonia were observed (Fig. 3). Supplementary Videos 1 and 2 provide axial CT images and 3D volume-rendered reconstructions of the thoracic skeletal abnormalities.

**Fig. 2:**
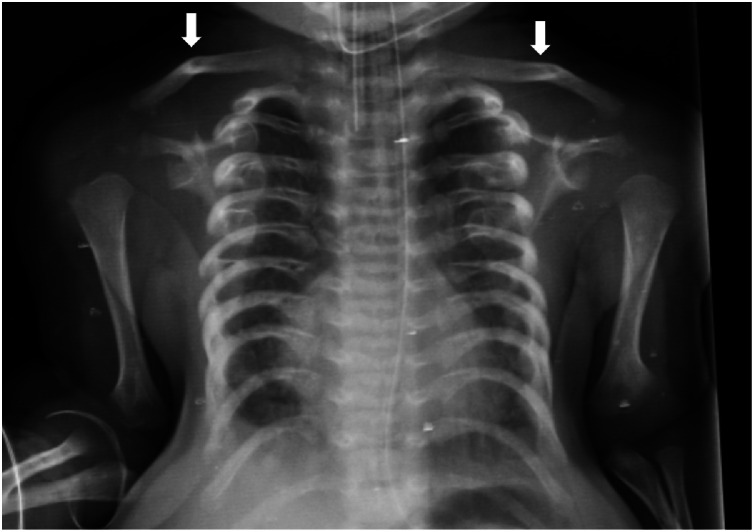
Frontal chest radiograph demonstrating an elongated thoracic cage with reduced transverse diameter, high-riding clavicles with a characteristic “handlebar” appearance (arrows), and irregular costochondral junctions.

**Fig. 3:**
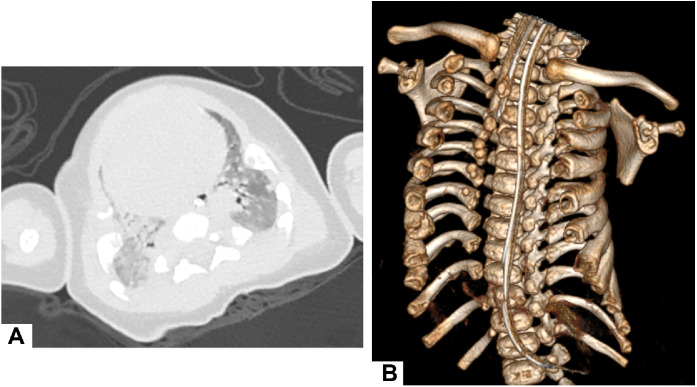
*(A)* Axial chest CT image (lung window) demonstrating bilateral pulmonary consolidations consistent with infectious pneumonia and *(B)* Three-dimensional CT reconstruction showing severe thoracic narrowing with short, horizontally oriented ribs, elevated clavicles, and irregular costochondral junctions.

Pelvic radiography revealed dysplastic acetabula with flattened acetabular roofs, trident-shaped acetabula, short and flared iliac bones, and shortened femora (Fig. 4). Radiographs of the extremities additionally demonstrated shortening of the long bones associated with metaphyseal irregularities (Fig. 5). The combination of thoracic and pelvic skeletal abnormalities was highly suggestive of a ciliopathy-related skeletal dysplasia, most consistent with asphyxiating thoracic dystrophy (Jeune syndrome).

**Fig. 4:**
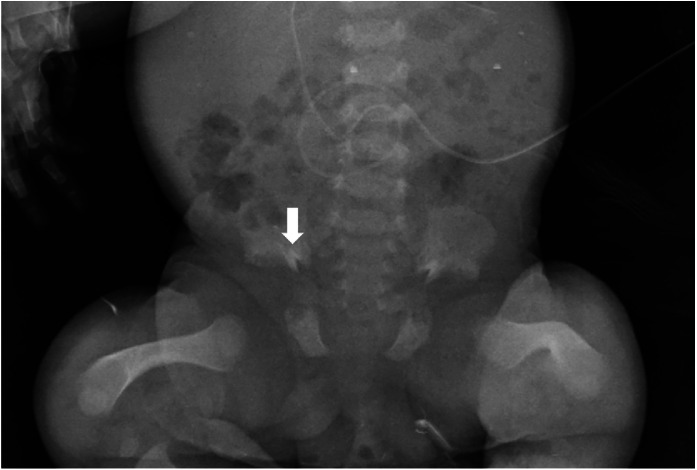
Frontal pelvic radiograph demonstrating characteristic skeletal abnormalities, including flattened acetabular roofs, trident-shaped acetabula (arrow), short and flared iliac bones, and shortened femora.

**Fig. 5:**
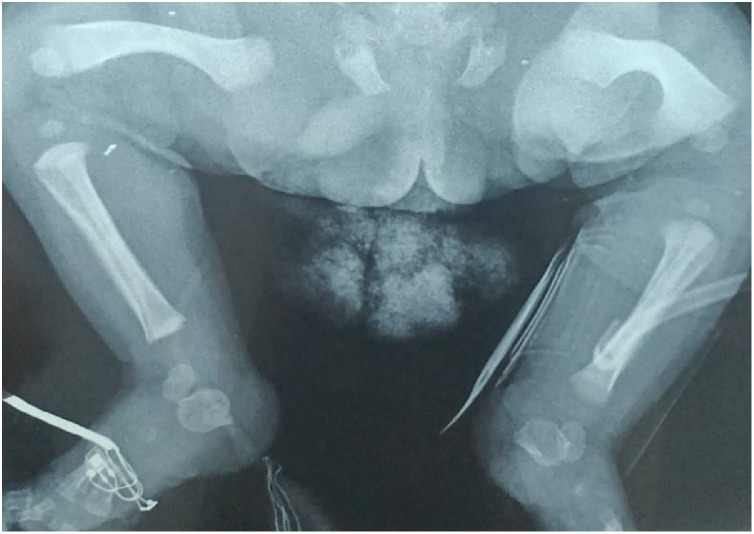
Radiograph of the extremities showing shortened long bones with metaphyseal irregularities.

The patient remained hospitalized in the neonatal intensive care unit for 7 days, requiring continuous invasive mechanical ventilation and receiving broad-spectrum antibiotic therapy for bilateral infectious pneumonia. Despite intensive supportive care, the respiratory condition progressively worsened due to severe thoracic restriction complicated by pulmonary infection. The neonate ultimately developed refractory respiratory failure and died during the hospital stay. No autopsy was performed.

## Discussion

Asphyxiating thoracic dystrophy (ATD), also referred to Jeune syndrome, is a rare autosomal recessive skeletal dysplasia associated with renal and hepatic anomalies [1]. It was first described in 1955 by Jeune et al. [2] in a newborn presenting with a narrow thoracic cavity and multiple cartilage anomalies, who died from respiratory insufficiency during the early neonatal period. The condition is estimated to occur in approximately 1 per 100,000-130,000 live births, with about 100 cases reported to date [2]. Genetically, ATD belongs to the group of ciliopathies and is caused by mutations in genes involved in primary cilia function and intraflagellar transport, most commonly *IFT80* and *DYNC2H1*, which disrupt normal skeletal development and contribute to the multisystem manifestations of the disease [3].

The diagnosis is established on the basis of clinical and radiographic findings, often identified on initial chest radiography [1]. Clinically, ATD is characterized by a small, constricted thoracic cage and variable degrees of limb shortening. Associated congenital anomalies include postaxial polydactyly of the hands and/or feet [1]. Typical radiographic features consist of a narrow, bell-shaped thorax with short, horizontally oriented ribs and irregular costochondral junctions, associated with elevated clavicles [3]. Additional findings include short iliac bones with a characteristic trident configuration of the acetabula, relatively short and broad long bones of the extremities, and hypoplastic phalanges of the hands and feet with cone-shaped epiphyses [3].

The clinical course of Jeune syndrome is highly variable, ranging from mild forms to fatal disease [4]. Respiratory distress and recurrent infections are common in the neonatal period and infancy and represent the main cause of early mortality, which may reach up to 60%-70% [4]. Such a presentation was observed in our patient, who developed severe neonatal respiratory distress with rapid progression to fatal failure. In patients who survive early childhood, thoracic growth may improve with subsequent enhancement of respiratory function [4]. However, late-onset complications may develop insidiously, particularly renal involvement progressing to renal failure, as well as hepatic dysfunction that may lead to cirrhosis [4].

The main differential diagnoses include Ellis–van Creveld syndrome, thoracolaryngopelvic dysplasia (Barnes syndrome), and short rib polydactyly syndromes [2]. Although Jeune syndrome shares phenotypic similarities with Ellis–van Creveld syndrome, thoracic involvement is typically more pronounced [2]. Radiographic features allow reliable distinction from Barnes syndrome—based on thoracic configuration, absence of laryngeal stenosis, and presence of iliac spurs in infancy—and from short rib polydactyly syndromes, which are usually associated with polydactyly and additional congenital anomalies [2].

Management of Jeune syndrome is primarily supportive [1]. Affected children frequently require intensive respiratory assistance during early childhood [2]. In cases of severe respiratory insufficiency, surgical intervention may be considered [2]. Various thoracic surgical approaches, including lateral thoracic expansion and chest wall reconstruction, have been attempted; however, no effective surgical technique has been established for neonatal patients [2]. Renal involvement may progress to renal failure, necessitating dialysis or renal transplantation [2]. Genetic counseling should be offered to the parents of affected individuals [2].

This case emphasizes the importance of considering Jeune syndrome in neonates presenting with severe respiratory distress. Early recognition, exclusion of alternative causes, and appropriate imaging are essential to establish the diagnosis and assess prognosis in this rare but life-threatening condition.

## Conclusion

Jeune syndrome is a rare skeletal dysplasia associated with significant neonatal morbidity and mortality due to thoracic insufficiency. Recognition of the characteristic radiographic features, including a narrow thoracic cage and associated skeletal abnormalities, allows prompt diagnosis of this condition. Early identification is essential to guide appropriate supportive management and to monitor for potential systemic complications, particularly renal and hepatic involvement.

## Author contributions

All authors contributed to this work. All authors have read and approved the final version of the manuscript.

## Ethical Approval

Our institution does not require ethical approval for reporting individual cases or case series.

## Patient consent

Written informed consent was obtained from the patient’s legal guardian for publication of this case report and accompanying images.

## Appendix Supplementary materials

Image, video 1 dummy alt text

Image, video 2 dummy alt text

Image, application 3
