# Supplementary material for: A rare cause of neonatal respiratory distress: Jeune syndrome
Source: Radiol Case Rep. 2026 Jun 11;21(9):3819–22. doi: 10.1016/j.radcr.2026.05.059 (PMC13276319; doi:10.1016/j.radcr.2026.05.059)
Supplement: Supplementary file 1 [file mmc1.docx]

**Supplementary Video legends :**

**Supplementary Video 1.** Axial thoracic CT images in lung window demonstrating severe thoracic narrowing and bilateral pulmonary consolidations.

**Supplementary Video 2.** Three-dimensional volume-rendered CT reconstruction showing characteristic thoracic skeletal abnormalities of Jeune syndrome.
